# Self-Management and Associated Factors among Patients with Non-Alcoholic Fatty Liver Disease: A Cross-Sectional Study

**DOI:** 10.3390/ijerph20010667

**Published:** 2022-12-30

**Authors:** Oh Young Kwon, Seung Up Kim, Sang Hoon Ahn, Yeonsoo Jang

**Affiliations:** 1 College of Nursing and the Brain Korea 21 FOUR Project, Yonsei University, Seoul 03722, Republic of Korea; 2 Department of Internal Medicine, Institute of Gastroenterology, College of Medicine, Yonsei University, Seoul 03722, Republic of Korea; 3 Mo-Im Kim Nursing Research Institute, College of Nursing, Yonsei University, Seoul 03722, Republic of Korea

**Keywords:** non-alcoholic fatty liver disease, self-efficacy, self-management, Korean

## Abstract

Self-management is critical and essential for controlling non-alcoholic fatty liver disease, delaying progression, and preventing complications. However, information about the self-management characteristics of this population is scarce. This study explores the characteristics and self-management levels and the factors associated with self-management in patients with non-alcoholic fatty liver disease in Korea. A convenience sample of 150 patients diagnosed with non-alcoholic fatty liver disease was recruited from April to November 2019. Demographics and clinical findings were collected, and self-management, self-efficacy, fatigue, and depressive symptoms were assessed using questionnaires. Multiple linear regression analysis was performed to examine the factors associated with self-management. Self-management levels were moderate (Mean = 3.4, SD = 0.61). Self-management differed significantly by age, sex, marital status, occupation, and health education experience. Self-efficacy (β = 0.074, *p* = 0.020) showed a significant association with self-management, which explained 25.0% of the variance after controlling for age, sex, marital status, health education experience, occupation, controlled attenuation parameter score, and body mass index. Self-efficacy is a critical determinant of self-management among patients with non-alcoholic fatty liver disease. The study findings could assist healthcare professionals in facilitating self-management compliance and developing multidisciplinary team-based interventions for sustainable self-management.

## 1. Introduction

Non-alcoholic fatty liver disease (NAFLD) is a common cause of chronic liver disease worldwide [1]. NAFLD comprises a broad spectrum of liver diseases, ranging from simple steatosis to steatohepatitis, which can potentially progress to liver cirrhosis and hepatocellular carcinoma [2]. Globally, the prevalence of NAFLD and its primary risk factors, including obesity and metabolic syndrome, are increasing rapidly [3]. In Asia, the prevalence of NAFLD is approximately 25%, similar to that in many Western countries [4]. South Korea has seen NAFLD prevalence rise to an estimated 31.5%, and the rate is even higher in individuals with obesity (56.7%), metabolic syndrome (63.2%), diabetes (58.2%), and hypertension (47%) [5].

NAFLD is defined as a hepatic fat accumulation of over 5% of the liver weight in the absence of excessive alcohol consumption (<20 g/day for men and 10 g/day for women) and other causes of liver fat [6,7]. NAFLD currently has no acceptable pharmacological agent; therefore, lifestyle management related to dietary habits and physical exercise is crucial to positive health outcomes [8]. It is recommended that patients with NAFLD gradually lose weight by reducing energy intake and increasing physical activity [7], and these guidelines have been supported by many intervention studies [9,10]. However, unlike most chronic diseases, NAFLD has no disease-specific symptoms until the condition turns serious. This characteristic makes it difficult to monitor and manage the disease continuously in daily life. Furthermore, it is challenging to change patients’ unhealthy lifestyles, and long-term maintenance of weight control is only achieved in a limited number of patients [6].

Self-management is the ability of individuals to be in charge of the symptoms, treatment, physical and psychological consequences, and lifestyle changes [11]. Self-management plays a crucial role in improving many chronic disease conditions. It allows patients to control or reduce the impact of the disease on their physical health status as they become better at coping with the associated psychosocial problems and managing their daily health-related behaviors [12]. Awareness of self-management has grown as a critical concern in the healthcare field as the prevalence of chronic diseases has increased, and healthcare professionals must focus on enabling patients to sustain self-management of their conditions. However, evidence suggests that well-structured interventions related to behavioral, physical, and dietary pattern modification have limited, rather than improved, patients’ ability to change and sustain healthy lifestyles [13]. Furthermore, health-related behaviors of NAFLD, such as weight control, can be affected by various factors, including age, sex, comorbidity, socioeconomic status, and acquired knowledge [14].

Additionally, self-efficacy, defined as one’s belief about their capabilities to perform behaviors for achieving a specific goal, is an essential component of self-management in chronic disease [15]. As a prerequisite to behavioral change, self-efficacy helps facilitate self-management and determine its sustainability when patients face obstacles caused by their disease. Moreover, it can lead to beneficial changes in health behaviors through goal setting and self-monitoring [16]. However, although it is crucial to improve self-management for patients, few studies have identified the association between self-efficacy and self-management of NAFLD.

Fatigue and depression must also be examined to improve self-management of NAFLD because they constitute a substantial health burden and are among the most disabling symptoms of many chronic diseases. As fatigue is accompanied by many chronic diseases [17], it may be associated with NAFLD, given that it is a chronic liver disease. Furthermore, 50–85% of patients reported fatigue, making it the most commonly reported symptom of chronic liver disease [18]. Some of these cases may be related to inflammation following NAFLD [19]. Additionally, depressive symptoms are often comorbid with chronic disease; thus, comorbidities can worsen health-related outcomes [20]. The prevalence of major depressive disorder among patients with NAFLD is 27.2%, which is higher than that of the general population [21].

An effective strategy for patients with NAFLD is required to improve their self-management of the disease. Some studies have targeted such patients, focusing on their diet and exercise to motivate and enhance their behaviors for weight control [8]. However, none have assessed the individual characteristics of NAFLD self-management. Therefore, to improve the self-management of NAFLD, identifying the psychological and sociodemographic factors related to NAFLD and investigating the relationships of self-management in this population is warranted.

The specific aims of this study are: (1) to explore the characteristics and level of self-management and (2) to examine the factors associated with the self-management of Koreans with NAFLD.

## 2. Materials and Methods

### 2.1. Study Design

This study was a secondary analysis of cross-sectional survey data from the research project of Self-Management using Augmented Reality Tutor for Liver (SMART-Liver): Self-Management Program of Non-Alcoholic Fatty Liver Patients. During the development stage of the program, we conducted several phased sub-studies, including qualitative research [22], the development of a self-management scale: NAFLD-SMQ [23], and identified associated factors of NAFLD self-management using NAFLD-SMQ.

### 2.2. Setting and Samples

This study was conducted at the outpatient clinic of the Liver Center of S Hospital, Seoul, South Korea. S Hospital is one of Korea’s oldest and largest university hospitals, with more than 3000 beds and approximately three million outpatients annually. The sample size was calculated using G* power software version 3.1 (Heinrich Heine University, Düsseldorf, Germany). The estimated sample size was 127, based on a medium effect size of 0.15, two-tailed α of 0.05, 12 candidate predictors (age, sex, marital status, occupation, education level, health education experience, comorbidity, body mass index, controlled attenuation parameter (CAP) score, self-efficacy, fatigue, and depression), and power of 0.8 samples. Finally, a sample size of 153 patients was estimated, considering a dropout rate of 20%. Eligible participants included in this study met the following criteria: (a) aged 19 or older, (b) diagnosed with NAFLD by elastography or ultrasonography for at least six months, and (c) the ability to read and write in Korean. Patients with the following conditions were excluded: hepatitis B virus carriers, liver cirrhosis, alcoholism, progressive cancer, and pregnancy.

### 2.3. Measures

#### 2.3.1. Self-Management

Self-management was assessed using the NAFLD Self-Management Questionnaire (NAFLD-SMQ) developed following the Individual and Family Self-Management Theory in patients with NAFLD in South Korea [23]. This scale consists of 22 items with six subdomains: medical treatment compliance (three items), management of medications and dietary supplements (two items), alcohol consumption management (three items), sleep management (three items), family support (four items), and lifestyle management (seven items). All items are rated on a five-point Likert scale ranging from 1 (*never*) to 5 (*always*). The mean scores were computed, with higher mean scores indicating a higher level of self-management. Psychometric analysis of this instrument demonstrated acceptable reliability and validity [24]. The reliability of this study was reported with a Cronbach’s alpha coefficient value of 0.84.

#### 2.3.2. Self-Efficacy

Self-efficacy was assessed using the 22-item Chronic Disease Self-Efficacy Scale–Korean Version (CDSES-K) [24], which is based on the Chronic Disease Self-Efficacy Scale (CDSES) developed by Lorig et al. [25]. Responses were provided on a ten-point Likert scale ranging from 1 (not at all confident) to 10 (totally confident), with higher scores indicating higher confidence. The Cronbach’s alpha coefficient was 0.91 in the original study and 0.93 in the Korean version. In this study, the reliability coefficient was 0.97.

#### 2.3.3. Fatigue

The subjective perception of fatigue was assessed using the Revised Piper Fatigue Scale—Korean Version [26], which was translated from the Revised Piper Fatigue Scale [27]. This scale consists of 22 items, with each item rated on a ten-point numeric rating scale ranging from 0 (none) to 10 (maximum intensity of the symptom). The scores for all items were summed, and the total scores were divided by the number of items. This scale provides an estimate of fatigue with 0 representing *none*, 1–3 as *mild*, 4–6 as *moderate*, and 7–10 as *severe* fatigue. For this scale, higher mean scores indicate higher perceived fatigue levels. The Cronbach’s alpha coefficient of the original scale was 0.97, and that of the Korean version was 0.96. The reliability coefficient was 0.97 in this study.

#### 2.3.4. Depressive Symptoms

Depressive symptoms were assessed using the Beck Depression Inventory I-Korean version [28], composed of 21 items. Each item is rated on a four-point Likert scale ranging from 0–3. The total score ranges from 0–63. Individuals with a total score between 0–13 were considered to have minimal depressive symptoms; scores from 14–19 were considered mild depressive symptoms, those from 20–28 were considered moderate depressive symptoms; and those from 29–63 were considered severe depressive symptoms. The Cronbach’s alpha coefficient was reported as 0.85 in a previous study [28], and in this study, internal consistency was 0.90.

#### 2.3.5. Demographic and Health-Related Characteristics

Demographic data (e.g., age, sex, marital status, education level, occupation, family income, and education experience related to liver disease) and clinical characteristics (i.e., treatment duration, comorbidities, and diagnostic methods of NAFLD) were collected to describe participant characteristics. NAFLD severity was assessed using elastography or ultrasonography. The CAP score was divided into three groups: Stage 1 (238–259), Stage 2 (260–292), and Stage 3 (≥293). Data on clinical characteristics were collected by reviewing medical records.

### 2.4. Ethical Consideration

This study was approved by the Institutional Review Board of Severance Hospital (IRB, No. 4-2018-1177). All participants provided informed consent. Data were maintained with confidentiality; disclosure of individuals’ information was warranted only to those with a legitimate interest, information was anonymized where possible, and disclosures were kept to the minimum necessary for the purpose.

### 2.5. Data Collection

The data were collected between April and November 2019. After IRB approval was obtained, healthcare providers at the clinic referred eligible participants (*N* = 177) to the researchers and confirmed their eligibility; the study purpose, methods, and confidentiality were explained. After obtaining informed consent, eligible participants completed the survey in a research room next to the outpatient clinic. The survey took an average of 15–20 min to complete. The researchers collected clinical data by reviewing the participants’ medical records.

### 2.6. Statistical Analysis

Data analysis was conducted using IBM SPSS Statistics for Windows (version 25.0; Armonk, NY, USA: IBM Corp.) with a significance level of *p* < 0.05. Descriptive statistics (i.e., mean, standard deviation [SD], frequency, and percentage) were computed to describe participants’ characteristics. To address the study’s first objective, we used a *t*-test or ANOVA to compare self-efficacy, fatigue, depressive symptoms, and self-management among patients grouped by demographics. Multiple linear regression was used to explore predictors (i.e., self-efficacy, fatigue, and depressive symptoms) of self-management, controlling for age, sex, marital status, occupation, education experience related to liver disease, and body mass index. We tested multicollinearity using tolerance values and Variation Inflation Factor (VIF); a tolerance > 0.1 and less than ten were regarded as acceptable [29]. We also reviewed the correlations between the independent and control variables to identify potential multicollinearity. Multicollinearity was considered if the correlation coefficient was more significant than 0.7.

## 3. Results

### 3.1. Participants’ Demographic and Health-Related Characteristics

We recruited 177 eligible patients from the clinic. Of 177, we selected only 150 participants who had completed the data for this study owing to missing data on critical variables. Table 1 presents the demographic characteristics; over half of the participants were men (59.3%), with a mean age of 49.0 (SD = 13.69), ranging between 19–86 years. Most participants were employed (70.7%), married (70.0%), and had graduated from high school or higher levels of education (92.7%).

Approximately 40% of participants consumed alcohol below the recommended amount in NAFLD guidelines; daily alcohol consumption was less than 30 g for men and 20 g for women [7]. Concerning health-related behaviors, over 50% reported exercising for approximately 40 min per day. Among participants, 44.7% reported sufficient sleep, and only 13.3% were current smokers. Most participants (84.7%) reported not receiving any education related to liver disease from hospitals or clinics.

Regarding the severity of NAFLD, 61.5% had a CAP score of more than 293 dB/m. Additionally, the average duration of NAFLD diagnosis was 28.0 months (SD = 33.93); most patients (91.3%) had at least one comorbidity, including hypertension (47.3%), hyperlipidemia (43.3%), or diabetes (28.7%). The mean body mass index was 28.2 (SD = 4.54), and 70% had a body mass index of 25 kg/m^2^ or over, which is considered obese among Koreans.

### 3.2. Levels of Self-Management

Table 2 shows the self-management levels in this study. The mean total of self-management was 3.4 (SD = 0.61), indicating a moderate level among the patients. In the self-management subdomains, the mean score for medical treatment compliance was the highest (mean = 4.1, SD = 0.87), followed by alcohol consumption management (mean = 4.0, SD = 1.14); however, the lifestyle management domain scored the lowest (mean = 3.0, SD = 0.91).

Differences in self-management in demographics and disease-related factors are presented in Table 3. Participants over 50 years had significantly higher levels of self-management (*p* = 0.005). Women had significantly higher self-management than men (*p* < 0.001), and the married group had higher self-management (*p* = 0.045). The unemployed group and participants with experience in liver disease education had significantly higher self-management scores (*p* < 0.001, *p* = 0.024).

### 3.3. Associated Factors of Self-Management

The correlation coefficients did not exceed 0.7, as shown in Table 4. The tolerance values (range: 0.6−0.9) and all VIF values were less than ten (range: 1.1−1.8); thus, no multicollinearity issues were found among the variables. The results of the multiple linear regression are presented in Table 5. Among the factors, only self-efficacy was significantly associated with self-management after controlling for age, sex, marital status, occupation, health education experience, CAP score, and body mass index (*β* = 0.074, *p* = 0.020). The model significantly predicted 25.0% of the variance in self-management (*p* < 0.001, adj. R^2^ = 0.207).

## 4. Discussion

This is the first study to examine levels of self-management using the NAFLD self-management scale among Koreans with NAFLD. The results include two key findings. First, the level of self-management was moderate, and “efforts to reduce alcohol consumption” were the highest self-management items for patients with NAFLD. Second, self-efficacy, occupation, and health education experience were significantly associated with self-management.

Overall, the level of self-management was moderate among the participants. In particular, the mean scores of medical treatment compliance and alcohol consumption management were higher than those of the other subdomains. In contrast, diet and exercise scores were the lowest mean scores related to sleep management and lifestyle management for weight control. Owing to a lack of study evidence related to the self-management of patients with NAFLD, it is difficult to compare the results of this study to those of previous studies. Nevertheless, the results of this study were similar to those in studies of patients with other liver diseases. Previous studies on adult patients with chronic hepatitis B reported a moderate score for hepatitis B self-management [30]. Similarly, adult patients with liver cirrhosis had a moderate level of self-management. They also reported the highest score for communication with physicians related to taking medicine and the lowest score for weight control or diet. However, the self-management level cannot be compared because of different self-management scales [31].

In contrast, patients with heart failure had low self-care and self-care maintenance habits, such as checking their weight or performing physical activity, as just over 7.0% of individuals reported good medical compliance [32]. Although different self-management scales were used, compared with other chronic diseases, the level of NAFLD self-management was similar or higher, but lifestyle management aspects were low. However, it might be insufficient to explain self-management characteristics in patients with NAFLD. Future studies should be conducted in diverse populations with NAFLD.

Another finding in this study highlighted that self-efficacy, occupation, and health education experience together explained a total of 25.5% of the variance in NAFLD self-management. Previous studies reported that self-efficacy was crucial in patients with other chronic diseases, such as diabetes or kidney disease, indicating that self-efficacy could help patients facilitate self-management behaviors [33,34]. A belief in self-efficacy can determine how people feel, think, motivate themselves, and behave toward their health [35]. Furthermore, information and feedback obtained about the results of their behaviors are a resource for self-efficacy.

Occupation was another significantly associated factor. In the work environment, work time and schedule constraints were identified as barriers to self-management behaviors related to diet and exercise [36,37]. Health-related education that facilitates self-management is essential to a successful self-management program. Previous studies showed that health education significantly improved the health outcomes of patients with chronic illnesses [38,39]. In particular, health education contributes to the enhancement of antecedents of behaviors, such as self-awareness, information, knowledge, skill, beliefs, attitudes, and values [40,41]. By providing health education, disease awareness and knowledge will be changed positively, which will lead to improvements in self-management. These results may support the importance of considering individual factors in developing interventions for the self-management of NAFLD patients. In addition, we cannot be certain that the participants of this study received the same form of health education. Effects of health education on the self-management in this population should be evaluated in future studies.

Although it was not an associated factor, there was a significant difference in levels of self-management according to the groups of patients aged 50 years or older. In general, the prevalence of NAFLD increases with age [14], and in adults under 50 years of age, it is more prevalent among men [42]. However, age was not presented as an associated factor of self-management in this study. Additionally, while marital status was not a predictor of self-management, it may be related to self-management. It is known that relationship status impacts family support because “being married” is considered the primary form of family support and a factor influencing self-management. Moreover, having a partner improves adherence to self-management behaviors in patients with chronic diseases [43,44]. Therefore, future studies should explore the relationship between these factors, such as age and marital status, and self-management among patients with NAFLD.

Besides these factors, fatigue was not a predictor of self-management but was significantly correlated with self-management. Fatigue was the most common symptom reported by patients with chronic liver disease in previous studies and appears significantly in NAFLD [17,19,45]. Most recent intervention studies related to fatigue were conducted to reduce the level of fatigue in cancer patients through self-management behaviors, such as physical activity, nutrition management, or cognitive behavior therapy [46,47]. However, fatigue is difficult to define as it involves complex interactions between biological, psychological, and behavioral processes [47], and research on the relationship between fatigue and self-management in chronic disease is still lacking. Additionally, longitudinal studies have examined the association of depression with the risk of NAFLD [48,49]. However, depression was not associated with self-management in this study, and the severity level of depression was low. The differences in depression severity may affect the relationships between self-management and depression. Therefore, further studies should be conducted to clarify this relationship, and research examining the mediating effect of self-efficacy between fatigue and self-management may be particularly useful.

Self-management, such as lifestyle modification, would be essential to achieving positive health outcomes in patients with NAFLD. However, NAFLD does not have severe symptoms unless it progresses to decompensated liver disease [1]. Owing to its progress, patients and even healthcare providers may not be concerned about the daily management of NAFLD. Understanding the factors affecting NAFLD self-management will help clinicians provide better strategies for improving disease conditions. Therefore, healthcare professionals should assess the level of NAFLD self-management and personalized factors affecting patients to achieve the goals for positive health outcomes in this population.

There are some limitations to this study. First, we included patients diagnosed with NAFLD by ultrasonography or elastography instead of liver biopsy; hence, mild steatosis may have been overlooked. However, ultrasonography and elastography are widely used to screen NAFLD epidemiologically in gastroenterology centers [6] because they are not invasive and are used with clinical status to diagnose NAFLD. Thus, the sensitivity for detecting steatosis would be acceptable. Second, our participants have a higher socioeconomic status than the general Korean population. For example, 68% of participants received a college or higher education. This may have influenced the results of this study. Third, although the sample size was acceptable in terms of statistical power analysis, we collected the data from one tertiary hospital in Korea. Therefore, the study findings have limited generalizability, and future studies should explore the factors affecting NAFLD among larger populations.

## 5. Conclusions

The level of self-management in this study was moderate. Self-management, self-efficacy, fatigue, and depressive symptoms significantly differ in individual characteristics regarding demographics and disease-related factors. Self-efficacy was an associated factor of self-management, adjusted for age, sex, marital status, occupation, education experience related to liver disease, and body mass index. These results may be clinically relevant as they reveal self-management factors in patients with NAFLD. Therefore, multidisciplinary team-based interventions should be based on self-efficacy to improve the self-management of NAFLD patients considering individual circumstances.

## Figures and Tables

**Table 1:** Demographic and health-related characteristics of participants (*N* = 150).

Characteristics	Category	Mean ± SD or n (%)
**Demographics**		
Age		49.0 ± 13.69
	<50	68 (45.3)
	≥50	82 (54.7)
Sex	Man	89 (59.3)
Marital status	Married	105 (70.0)
Occupation	Yes	106 (70.7)
Education level	<High school	10 (7.3)
(n = 149)	High school	37 (24.7)
	≥College	102 (68.0)
House income (USD)	≤2000	19 (12.9)
(n = 147)	2001–4000	51 (34.7)
	>4001	77 (52.4)
**Health-related**		
Sleep time (an hour per day)		6.3 ± 1.29
Sleep sufficiency	Yes	67 (44.7)
Alcohol consumption	Yes	59 (39.3)
Smoking	Yes	20 (13.3)
Exercise	Yes	90 (60.0)
Walking	Yes	79 (52.6)
Sedentary time (an hour per day)		6.8 ± 3.62
Health education experience	Yes	23 (15.3)
Length of diagnosis (months)		28.0 ± 33.93
Comorbidity ^a^	Yes	137 (91.3)
	Hypertension	71 (47.3)
	Hyperlipidemia	65 (43.3)
	Diabetes	43 (28.7)
	Others	92 (61.3)
CAP ^b^ score (dB/m)		307.9 ± 39.40
CAP score stage ^c^	Normal (<238)	4 (2.8)
(n = 143)	1 (238–259 dB/m)	11 (7.7)
	2 (260–292 dB/m)	40 (28.0)
	3 (≥293 dB/m)	88 (61.5)
Body mass index (kg/m^2^)		28.2 ± 4.54
	Normal (<23.0)	9 (6.0)
	Overweight (23.0–24.9)	36 (24.0)
	Obese (≥25.0)	105 (70.0)
**Psychosocial**		
Self-efficacy		7.0 ± 1.63
Fatigue		4.6 ± 2.05
Depression		10.9 ± 8.18

*Note*. ^a^ = multiple responses; ^b^ = controlled attenuation parameter; ^c^ = Patients diagnosed by ultrasonography excluded.

**Table 2:** The level of self-management among participants with NAFLD (*N* = 150).

Subdomains with Items of Self-Management	Mean ± SD
**Total self-management**	3.4 ± 0.61
**Medical treatment compliance**	4.1 ± 0.87
Periodic checkups to prevent fatty liver progression	4.3 ± 0.85
Periodic checkups for disease management	4.2 ± 1.15
Communication with a healthcare provider about taking medication	3.8 ± 1.21
**Alcohol consumption management**	4.0 ± 1.14
Efforts to reduce alcohol consumption	4.5 ± 0.83
Management of alcohol consumption	3.9 ± 1.47
Setting the amount of alcohol consumption	3.6 ± 1.54
**Family support**	3.4 ± 0.93
Hospital checkups	3.7 ± 1.25
Use of medication	3.6 ± 1.22
Intake of health supplements	3.3 ± 1.26
Exercise	3.1 ± 1.25
**Management of medications and dietary supplement** **s**	3.2 ± 1.15
Confirmation of medication compliance	3.7 ± 1.30
Consultation with healthcare providers	2.8 ± 1.38
**Sleep management**	3.1 ± 0.99
Improving sleep disturbances	3.5 ± 1.17
Sufficient sleep	2.9 ± 1.23
Setting a time to sleep	2.8 ± 1.26
**Lifestyle management**	3.0 ± 0.91
Increase in daily activities	3.4 ± 1.09
Control of food intake, carbohydrates, and sugar	3.4 ± 1.07
Planning of number of workouts or workout time	3.1 ± 1.26
Weight loss	3.0 ± 1.23
Regular exercise	3.0 ± 1.51
Calorie, carbohydrate, and sugar intake	2.8 ± 1.31
Daily food intake and frequency	2.5 ± 1.30

*Note.* NAFLD = non-alcoholic fatty liver disease.

**Table 3:** Differences in self-management by demographic and disease-related factors (*N* = 150).

Variable	Category	Self-Management		
		Mean (SD)	F/t	*p*
Age	<50	3.2 (0.53)	−2.83	0.005
	≥50	3.5 (0.65)		
Sex	Man	3.2 (0.55)	4.10	<0.001
	Woman	3.6 (0.63)		
Marital status	Married	3.5 (0.63)	−2.02	0.045
	Others	3.2 (0.54)		
Occupation	Yes	3.3 (0.52)	3.96	<0.001
	No	3.7 (0.69)		
Education level	<High school	3.5 (0.93)	0.828	0.439
	High school	3.5 (0.63)		
	≥College	3.3 (0.56)		
Health education experience	Yes	3.7 (0.62)	−2.28	0.024
	No	3.3 (0.60)		
Comorbidity	Yes	3.4 (0.60)	0.84	0.403
	No	3.5 (0.79)		
CAP ^a^ score stage	Normal	3.5 (0.50)	1.60	0.193
	1	3.7 (0.63)		
	2	3.5 (0.70)		
	3	3.3 (0.57)		
Body mass index	<23.0	3.8 (0.82)	2.52	0.084
(kg/m^2^)	23.0–24.9	3.4 (0.64)		
	≥25.0	3.3 (0.58)		

*Note.* ^a^ = controlled attenuation parameter.

**Table 4:** Correlation matrix.

Variables	1	2	3	4	5
1. Age					
2. Body mass index	−0.28 **	-			
3. Self-efficacy	0.20 *	−0.17 *	-		
4. Fatigue	−0.30 ***	0.21 *	−0.40 **	-	
5. Depression	−0.13	0.27 **	−0.45 **	0.65 **	
6. Self-management	0.24 **	−0.19 *	0.27 **	−0.22 **	−0.14

*Note.* *: *p* < 0.05; **: *p* < 0.01; ***: *p* < 0.001.

**Table 5:** Factors associated with self-management among patients with NAFLD.

Variable	Self-Management				
	*β*	*SE*	Std. *β*	T	*p*
Age	−0.002	<0.01	−0.039	−0.41	0.682
Sex ^a^	−0.139	0.12	−0.111	−1.15	0.254
Marital status	0.136	0.12	0.102	1.18	0.239
Occupation ^b^	−0.359	0.13	−0.268	−2.81	0.006
Health education experience ^b^	0.276	0.13	0.163	2.17	0.032
Body mass index	−0.011	0.01	−0.084	−1.08	0.281
Self-efficacy	0.074	0.03	0.196	2.36	0.020
Fatigue	−0.023	0.03	−0.078	−0.95	0.344

*Note.* ^a^ = reference: woman; ^b^ = reference: no; NAFLD = non-alcoholic fatty liver disease; *β = unstandardized coefficient*; Std. *β = standardized coefficient.*

## Data Availability

The data used in this study are available from the corresponding author upon reasonable request.
